# The adrenal gland and primary aldosteronism: anatomy, steroidogenesis, regulation, and genetic insights

**DOI:** 10.3389/fendo.2025.1736534

**Published:** 2026-01-12

**Authors:** Kha Chin Long, Elena Aisha Azizan

**Affiliations:** 1 Department of Medicine, Faculty of Medicine, The National University of Malaysia (UKM), Kuala Lumpur, Malaysia; 2 Research Center, Hospital Tunku Ampuan Besar Tuanku Aishah Rohani, Universiti Kebangsaan Malaysia Specialist Children’s Hospital, Kuala Lumpur, Malaysia; 3 William Harvey Research Institute, Queen Mary University of London, London, United Kingdom

**Keywords:** adrenal cell proliferation, adrenal gland, aldosterone dysregulation, aldosterone, primary aldosteronism

## Abstract

The adrenal glands play an essential role in maintaining homeostasis through the secretion of steroid hormones. Among these, aldosterone, a mineralocorticoid produced in the zona glomerulosa (ZG) of the adrenal cortex, regulates fluid balance and blood pressure. This review summarizes the current knowledge of adrenal anatomy, aldosterone biosynthesis and regulation, and the pathophysiology and genetic landscape of primary aldosteronism (PA), a common form of secondary hypertension. Advances in next-generation sequencing have enabled the discovery of novel somatic and germline mutations underlying PA, elucidating their roles in abnormal aldosterone production and adrenal cell proliferation. Understanding the molecular basis of aldosterone dysregulation provides critical insights into PA subtypes, informing the development of improved diagnostic and therapeutic strategies.

## 1 Introduction

The adrenal glands are part of the endocrine system that secretes hormones to regulate vital body functions, such as blood pressure, the body’s stress response, metabolism, immune function, and development of sexual characteristics. The anatomy of the adrenal gland was first described by Bartolomeo Eustachio in 1564 (1). However, the functional role of the adrenal gland was only accurately defined approximately three decades later in 1855 by Thomas Addison (2). Addison reported the autopsy findings of 11 subjects, described as having “diseased” adrenal glands (bilaterally), with clinical manifestations of asthenia, lethargy, progressive weight decline, anemia, cardiac insufficiency, and hyperpigmentation of the skin. A year later, Charles Brown-Sequard further demonstrated that the adrenal glands are important for survival in animals (3). He showed that removal of both adrenals caused lethal effects in dogs, cats, and guinea pigs and concluded that the cause of death in these animals was due to a lack of adrenal hormone secretion.

## 2 Anatomy of the adrenal gland

**Figure 1:**
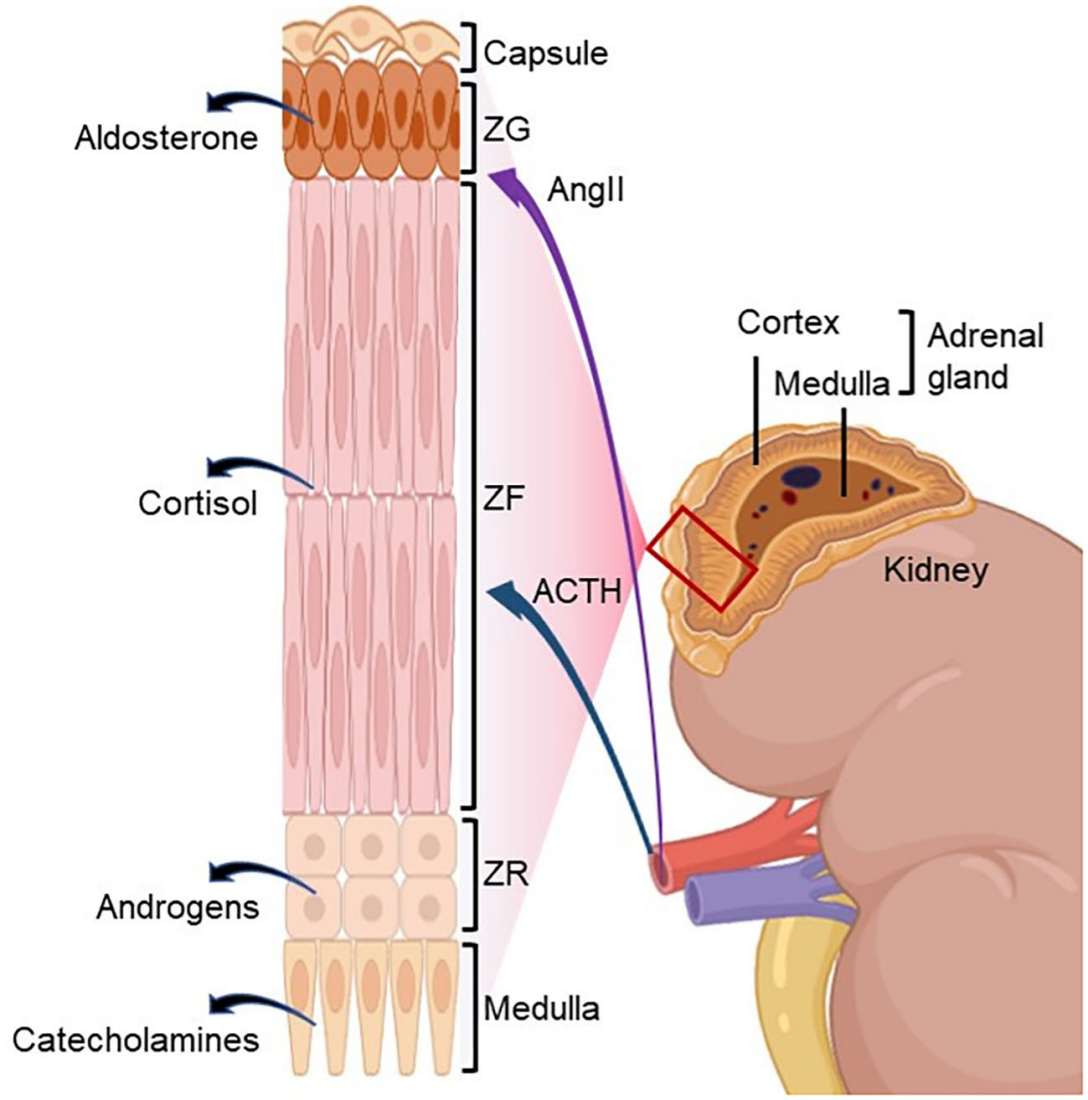
Adrenal zonation with primary regulators of steroidogenesis. The human adrenal gland is composed of an outer cortex and an inner medulla. The adrenal cortex is further subdivided into three distinct functional zones: the zona glomerulosa (ZG), zona fasciculata (ZF), and zona reticularis (ZR). The ZG produces the mineralocorticoid aldosterone, primarily regulated by angiotensin II (AngII). The ZF synthesizes the glucocorticoid cortisol under the main control of adrenocorticotropic hormone (ACTH). The ZR secretes adrenal androgens also under the control of ACTH. The adrenal medulla produces catecholamines under the control of the sympathetic nervous system. Created with BioRender.com.

The adrenal glands are paired endocrine organs that lie bilaterally above the kidneys. In adults, the adrenal glands exhibit morphological asymmetry; the right adrenal gland has a pyramid-like morphology, whereas the left adrenal gland has a more crescentic configuration. The average weight of the adrenal gland is 4 g, measuring approximately 2 cm wide, 5 cm long, and 1 cm thick (4). The human adrenal glands consist of an outer cortex and an inner medulla. The adrenal cortex can be further differentiated into three major zones: zona glomerulosa (ZG), zona fasciculata (ZF), and zona reticularis (ZR) (**Figure 1**; **Table 1**).

**Table 1:** Zones of the adrenal gland and associated cellular characteristic with the main hormone secreted of each zone.

Zone	Cellular characteristic	Main hormones secreted
Zona glomerulosa (ZG)	Small, rounded cells organized in clusters, compact nuclei	Mineralocorticoids (aldosterone)
Zona fasciculata (ZF)	Large, polyhedral cells organized in columns, with vacuolated cytoplasm due to high intracellular lipid content	Glucocorticoids (cortisol)
Zona reticularis (ZR)	Cells organized in tightly packed columns, with sparse lipid	Androgens (DHEA and androstenedione)
Adrenal medulla	Irregular trabeculae of polygonal cells, with sparse lipid	Catecholamines (norepinephrine and epinephrine)

The ZG is located directly under the capsule, occupying approximately 15% of the adrenal cortex. Its thickness may be reduced in individuals consuming a high sodium diet (5–7). The ZG produces mineralocorticoids, mainly aldosterone, to maintain the fluid and electrolyte balance. The ZF occupies approximately 75% of the adrenal cortex and is responsible for the synthesis of glucocorticoids, primarily cortisol, which is essential for regulating gluconeogenesis and glycogenesis (8–11). The innermost layer of the adrenal cortex is the ZR, which encircles the adrenal medulla positioned at the central core of the adrenal gland. The ZR secretes androgens, including dehydroepiandrosterone (DHEA) and dehydroepiandrosterone sulfate (DHEAS) (4, 12). **Table 1** summarizes the cellular characteristics and main hormonal profiles of the adrenal glands.

## 3 Aldosterone biosynthesis and regulation

**Figure 2:**
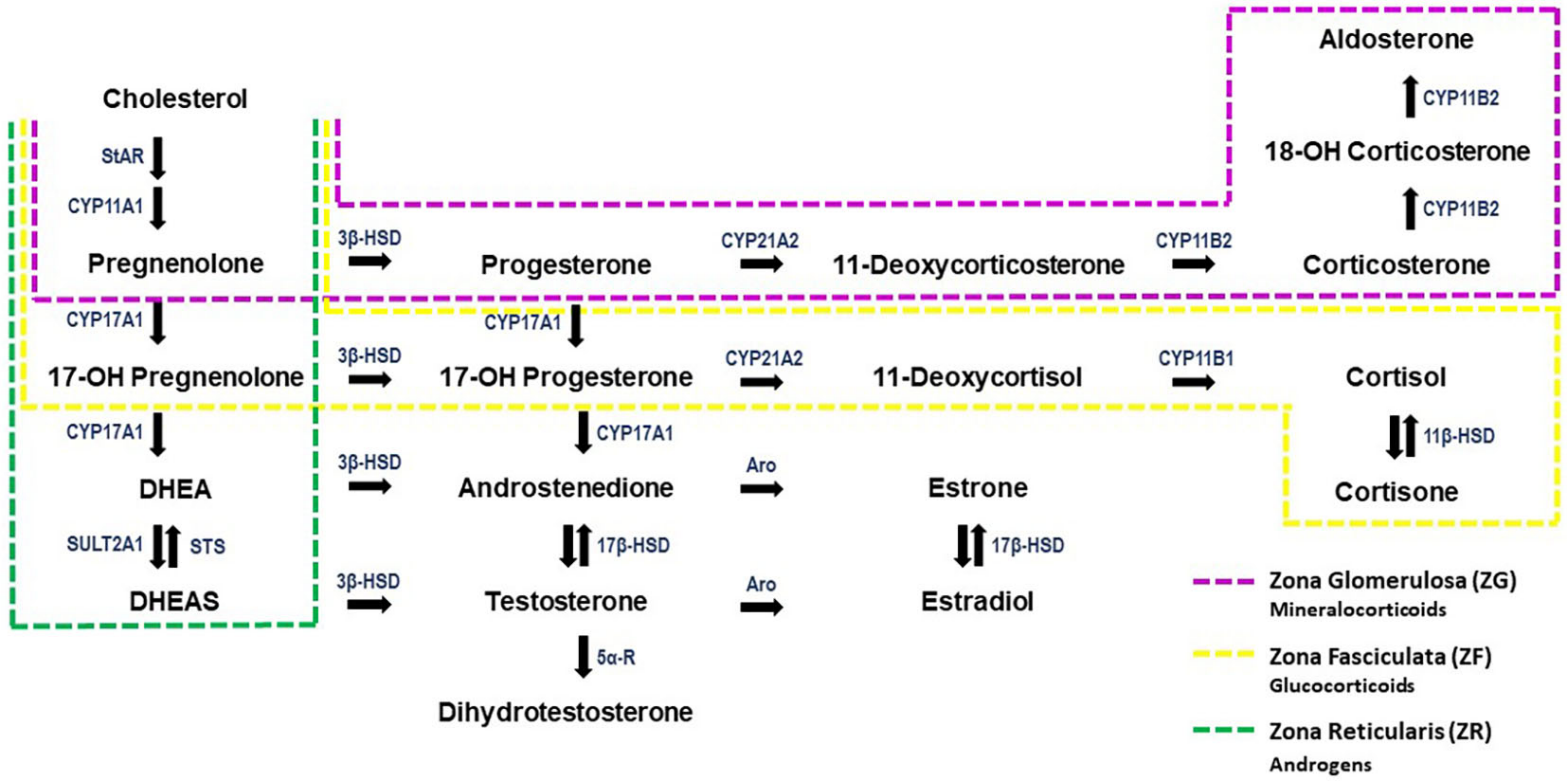
The steroidogenesis pathway. Steroid hormones are indicated in black, enzymes are denoted in blue. The regions of the adrenal cortex in which each hormone is produced is indicated by the dashed, coloured boxes. DHEA, Dehydroepiandrosterone; DHEAS, Dehydroepiandrosterone Sulfate; Aro, Aromatase; StAR, Steroidogenic acute regulatory protein; STS, Steroid Sulfatase; SULT2A1, Steroid Sulfotransferase 2A1; 5α-R, 5α-Reductase. Image modified from Storbeck et al. (2019) (17) and Kater et al. (2022) (18).

The mineralocorticoid aldosterone is synthesized in the adrenal gland, precisely in the ZG of the adrenal cortex. Aldosterone production is a dynamic process, inextricably bound to *de novo* synthesis, due to the inability of the adrenal glands to store hormones once they are produced and for immediate release (13–16). The biochemical pathways involved in adrenal steroidogenesis are illustrated in **Figure 2**.

Cholesterol is the precursor for all steroid hormones’ synthesis, including aldosterone. To initiate steroidogenesis, intracellular cholesterol is mobilized from the outer to the inner mitochondria membrane mediated by Steroidogenic Acute Regulatory Protein (StAR) (19), for conversion to pregnenolone by CYP11A1. Pregnenolone is then passively diffused into the cytoplasm and converted to progesterone by the type II isozyme 3β-HSD (HSD3B2). Progesterone is hydroxylated to 11-deoxycorticosterone by the enzyme CYP21A. Finally, in the mitochondria of ZG, aldosterone is converted from 11-deoxycorticosterone via three sequential enzymatic reactions catalyzed by aldosterone synthase (CYP11B2)—11-hydroxylation, 18-hydroxylation, and 18-oxidation (20).

The *CYP11B2* gene is 95% identical in the coding regions and approximately 90% identical in the intron regions with *CYP11B1* that encodes for 11-hydroxylase, which is responsible for the final catalysis of 11-deoxycortisol to cortisol (21). Despite the high homology between *CYP11B1* and *CYP11B2*, their 5′ promoter regions differ markedly, allowing for the distinct regulation of cortisol and aldosterone synthesis, primarily by adrenocorticotropic hormone (ACTH) and angiotensin II (AngII), respectively (22, 23). The regulation of aldosterone is essential for retaining sodium when salt intake is low, thereby maintaining blood volume and blood pressure. Other factors or physiological agonists that facilitate the regulation of aldosterone secretion are potassium (K^+^) and ACTH, whereas dopamine, atrial natriuretic peptide, and heparin act as antagonists of aldosterone secretion (4).

## 4 Mechanisms of aldosterone regulation

Aldosterone secretion is primarily regulated by three key mechanisms: (i) the renin–angiotensin–aldosterone system (RAAS), (ii) circulating potassium concentration, and (iii) ACTH. The physiological pathways through which these factors modulate aldosterone synthesis and release are described below.

### 4.1 The renin–angiotensin–aldosterone system

The RAAS is the primary regulator of aldosterone production under normal physiological conditions. This system is vital for maintaining blood pressure and volume. RAAS is activated when the baroreceptors in the carotid sinus detect a decrease in intravascular volume and pressure, resulting in renin release from juxtaglomerular cells in the kidney by cleavage of its inactive precursor prorenin (24). Renin catalyzes the cleavage of angiotensinogen to angiotensin I (AngI), which is further converted to AngII by the angiotensin-converting enzyme (ACE). AngII affects blood volume and pressure through (i) increasing aldosterone secretion from the ZG by increasing *CYP11B2* transcription, (ii) constricting vascular smooth muscle to maintain blood pressure, (iii) releasing norepinephrine and epinephrine from the adrenal medulla, (iv) increasing central sympathetic outflow, and (v) promoting the release of vasopressin (4).

AngII mediates both acute and chronic stimulation of aldosterone synthesis. Under acute conditions, AngII enhances cholesterol transport to the inner mitochondrial membrane by upregulating the expression and phosphorylation of StAR, thereby stimulating the rapid synthesis of aldosterone. Chronic or long-term aldosterone production is primarily mediated by the upregulation of *CYP11B2* expression (25). Binding of AngII to the type 1 AngII (AT1) receptor, a G-protein-coupled receptor, activates phospholipase C (PLC) to hydrolyze phosphatidylinositol 4,5-bisphosphate (PIP2) to diacylglycerol (DAG) and inositol 1,4,5-trisphosphate (IP3). IP3 increases intracellular calcium concentration, resulting in the phosphorylation of calcium/calmodulin (CaM)-dependent protein kinase I/II (CaMK) and activation of transcription factor-1 (ATF-1), ATF-2, and cAMP-response-element-binding (CREB) protein. CREB protein binds to the 5′ untranslated region of the *CYP11B2* gene, thereby promoting its transcriptional activation (26).

### 4.2 Potassium concentration

The synthesis of aldosterone is extremely sensitive to modulation of K^+^ concentration; changes in 5%–8% of circulating K^+^ concentration can alter serum aldosterone levels by 40%–50% (27). Aldosterone production is stimulated by elevated K^+^ levels, which aid in maintaining K^+^ homeostasis. The actions of AngII and K^+^ work in concert, with K^+^ determining the amount of aldosterone produced in response to AngII (28). An increase in K^+^ concentration depolarizes the ZG cell membrane, opening the voltage-dependent L-type and T-type calcium (Ca^2+^) channels and elevating intracellular Ca^2+^ levels. This activates CaM and CaM-dependent kinases, which, in turn, phosphorylate transcription factors such as ATF-1, ATF-2, and CREB protein. These phosphorylated factors thus enhance the transcription of *CYP11B2* (29).

### 4.3 Adrenocorticotropic hormone

ACTH also plays a role in the stimulation of aldosterone secretion. The interaction between ACTH and G-protein-coupled receptors in the ZG activates adenylate cyclase, thus elevating cAMP and activating protein kinase A (PKA) (30, 31). The *CYP11B2* transcription is increased in a cAMP-responsive manner (23). However, studies in both human and animal models have found that high doses of ACTH suppress *CYP11B2* expression and aldosterone production (32–34). The mechanism underlying chronic inhibition is unclear—some suggest that the expression of AngII receptors in ZG cells may be downregulated by cAMP (35, 36) or that ACTH may transform ZG cells to ZF cells or divert precursors from the mineralocorticoid to the glucocorticoid pathway (8, 37, 38). This pattern of inhibition with high hormone levels is not unique to ACTH; similar desensitization is observed in other pituitary–target gland axes—for example, suppression of testicular testosterone production with high doses of LH or prolonged LH-RH stimulation—and suggests a shared regulatory principle across endocrine systems (39).

## 5 Primary aldosteronism

Hypertension is one of the most prevalent chronic medical disorders and the leading contributor to cardiovascular-related morbidity and mortality, affecting over 1 billion adults globally (40). While the majority of patients are reported with essential hypertension, primary aldosteronism (PA) appears to be the most common form of secondary hypertension, accounting for 3.2% to 14% of patients in primary care (41–45), increasing to 10% to 20% of patients referred to specialist care (42, 43, 46). However, notable challenges in the screening and diagnosis of PA could lead to the prevalence of this disease being markedly underestimated (47–49).

PA is characterized by the autonomous secretion of aldosterone from one or both adrenal glands, which is not under the control of RAAS, as the secretion of renin is suppressed. This results in sodium and water retention, consequent elevation of blood pressure, and, occasionally, in a minority of cases, hypokalemia, ultimately contributing to cardiovascular damage (50). This is mainly through the increased expression of ENaC and Na^+^/K^+^ ATPase, which leads to increased Na^+^ reabsorption, causing a negative luminal potential and increased K^+^ secretion into urine (14, 51). Aldosterone can also cause metabolic alkalosis, which favors K^+^ loss (52). To note, individuals with PA have more severe cardiovascular morbidity and mortality than those with essential hypertension, as excess aldosterone has detrimental effects on the heart, regardless of blood pressure levels (53, 54). Thus, the current recommendation by the Endocrine Society is that all individuals with hypertension be screened for PA by measuring serum or plasma aldosterone concentration and plasma renin concentration or activity to calculate the aldosterone-to-renin ratio (ARR) (55).

PA screening requires concurrent measurement of serum or plasma aldosterone concentration and plasma renin obtained in the morning with the patient in a seated position to calculate the ARR. The serum potassium levels should also be measured simultaneously to prevent misinterpretation of aldosterone levels (55). In most cases, patients with high ARR undergo PA confirmatory tests such as (i) the saline infusion test (SIT), (ii) the oral salt suppression test, (iii) the captopril challenge test, or (iv) the fludrocortisone suppression test (50). To further determine the PA lateralization type, recent guidelines recommend the use of computed tomography (CT) in conjunction with adrenal vein sampling (AVS) before deciding on the appropriate treatment approach, whether surgical or medical (55–57). Patients with unilateral PA have the potential to achieve a complete cure with adrenalectomy, especially when diagnosed early, whereas subjects with bilateral PA will need to undergo lifelong medical treatment with mineralocorticoid receptor antagonists (MRAs) such as spironolactone or eplerenone.

In the Primary Aldosteronism Surgery Outcome (PASO) study, the postoperative outcomes in patients with unilateral PA who undergo surgical intervention are further classified according to the clinical and biochemical outcomes (50, 58–61). The clinical and biochemical outcomes were classified into complete (cure), partial (improvement), and absent (failure) success based on blood pressure, the number of antihypertensive medications, plasma potassium concentration, plasma aldosterone concentration, and plasma renin concentration (58). Similarly, the Primary Aldosteronism Medical Treatment Outcome (PAMO) provides consensus on the definition of complete, partial, or absent biochemical and clinical outcomes of medical treatment of PA (62). The PAMO criteria comprise three clinical and three biochemical response categories, and closely parallel the PASO criteria, with the key distinction that PAMO incorporates unsuppressed renin as a requirement for defining a complete biochemical response (62).

## 6 Subtypes of PA

PA is commonly subtyped based on the lateralization of aldosterone production, that is, unilateral PA or bilateral PA. In 2021, a consensus on the histopathology classification of PA, known as HISTALDO, was established to provide histological diagnostic criteria for unilateral PA. This classification employs CYP11B2 immunohistochemistry (IHC) analysis of adrenalectomy samples for evaluation (63). The HISTALDO sub-categorizes the adrenal cortical lesions found in unilateral PA as follows: (i) aldosterone-producing adenoma (APA), benign hormone-secreting tumors with a diameter of ≥10 mm; (ii) aldosterone-producing nodule (APN), lesions <10 mm visibly discerned by hematoxylin–eosin staining; (iii) aldosterone-producing micronodule (APM), previously referred to as aldosterone-producing cell cluster (APCC), lesion <10 mm composed of ZG cells not discernable by hematoxylin–eosin staining; (iv) multiple aldosterone-producing nodules (MAPN) or multiple aldosterone-producing micronodules (MAPM), formally known as micronodular hyperplasia; (v) aldosterone-producing diffuse hyperplasia (APDH); and (vi) aldosterone-producing adrenocortical carcinoma (APACC) (63).

A major cause of unilateral PA is APA, also known as Conn’s syndrome, which accounts for approximately 30% of all PA cases (64). Varying degrees of diffuse or nodular hyperplasia are frequently observed in the ipsilateral adrenal cortex of APA (65). In recent years, significant progress has been achieved in elucidating the genetic underpinnings of APAs. The advent of high-throughput next-generation sequencing technologies has enabled comprehensive comparisons of whole-exome genetic variations between germline DNA and somatic DNA derived from APAs, thereby substantially enhancing our understanding of PA pathophysiology. Many somatic gene mutations—*KCNJ5* (66), *CACNA1D* (67, 68), *ATP1A1* (69), *ATP2B3* (69), *CTNNB1* (70, 71), *CLCN2* (72), *CACNA1H* (73), *GNA11/Q* (74), *CADM1* (75), *SLC30A1* (76), and *MCOLN3* (77)—have been found in APAs. **Table 2** summarizes the somatic mutations identified in APAs, along with their associated pathophysiological mechanisms and phenotypic characteristics.

**Table 2:** Genetic causes of APAs.

Gene OMIM	Encoded protein	Year first reported (reference)	Mechanism in PA pathophysiology and phenotypes
*KCNJ5* 600734	Potassium channel inwardly rectifying channel subfamily J member 5; GIRK4 inwardly rectifying potassium channel Kir3.4	2011 (66)	Dysfunction of K^+^ GIRK4 (Kir3.4) potassium channel, abnormal Na^+^ permeability, increased Ca^2+^ influx, increased aldosterone production; deregulated cell growth. Female, younger, and East Asian patient dominance; larger, often ≥15–20 mm APA, elevated aldosterone production and pronounced hypokalemia (78–81).
*CACNA1D* 114206	Calcium channel voltage-dependent L-type alpha1D subunit	2013 (67, 68)	Ca^2+^ channel depolarization, increased Ca^2+^ signaling, and increased aldosterone production. Male and black ethnicity dominance; size typically <20 mm (reduced APA size relative to *KCNJ5* mutant APAs) (78, 82).
*ATP1A1* 182310	ATPase Na^+^/K^+^ transporting alpha 1 polypeptide	2013 (69)	Induced membrane depolarization, increased Ca^2+^ influx, increased aldosterone production; deregulated cell growth. Male dominance; size typically <20 mm (reduced APA size relative to *KCNJ5* mutant APAs), high aldosterone level and hypokalemia (78, 83).
*ATP2B3* 300014	ATPase Ca^2+^ transporting plasma membrane 3	2013 (69)	Induced membrane depolarization, increased Ca^2+^ influx, increased aldosterone production. Male dominance; high aldosterone level and severe hypokalemia (78, 83).
*CTNNB1* 116806	β-catenin	2008 (70) 2015 (71)	Stimulated aldosterone production, modulated cell growth. Female dominance; linked to pregnancy and menopause; elevated *LHCGR* and *GNRHR* gene expression (71, 84).
*CLCN2* 600570	Voltage-gated chloride channel 2	2018 (72)	Ca^2+^ channel depolarization, increased Ca^2+^ signaling, and increased aldosterone production. Young dominance; smaller size of APA compared to *KCNJ5* tumor (85, 86).
*CACNA1H* 607904	Calcium channel voltage-dependent T-type alpha 1H subunit; Ca_v_3.2	2020 (73)	Ca^2+^ channel depolarization, increased Ca^2+^ signaling, and increased aldosterone production. Heterogeneous expression of *CYP11B2* within the tumor tissue; *CACNA1H* variant detected only in the CYP11B2-positive region of the tumor (73).
*GNA11/Q* 139313 600998	Guanine nucleotide-binding protein alpha 11/Q polypeptide; G protein subunit alpha 11/Q; Gα11/q	2021 (74)	Autonomous aldosterone production, modulated cell growth. Female dominance; linked to pregnancy and menopause; elevated *LHCGR* gene expression; hyperplasia of adjacent ZG and low *CYP11B1* expression (74).
*CADM1* 605686	Cell adhesion molecule 1; neuronal cell adhesion	2023 (75)	Inhibition of gap junction and modulation of biology rhythms, periodic aldosterone secretion. APA with abundant *CYP11B2* expression; CADM1 expression in the adjacent ZG and adrenal medulla (75).
*SLC30A1* 609521	Solute carrier family 30 member 1, Zink efflux transporter 1	2023 (76)	Increased Zn^2+^ permeability, increased Na^2+^ conduct, increased Ca^2+^ signaling, increased aldosterone production. APA with abundance *CYP11B2* expression; *SLC30A1* expression in adjacent adrenal tissue (76).
*MCOLN3* 607400	Mucolipin-3, transient receptor potential cation channel, mucolipin subfamily, member 3 (TRPML3)	2025 (77)	Abnormal Ca^2+^ influx or abnormal Na^+^ permeability, thus increased Ca^2+^ influx, increased aldosterone production. Male and old age dominance; APA size between 12 and 17 mm, APA exhibited sparse MCOLN3 expression compared to adjacent ZG.

Bilateral PA, also known as idiopathic hyperaldosteronism (IHA), is the most common form of all PA occurrences, accounting for nearly two-thirds of cases. In bilateral PA, CT findings commonly find adrenal hyperplasia, which can be diffuse or nodular, and APAs that are present bilaterally (65).

Familial forms of PA are relatively uncommon, accounting for approximately 1%–5% of all cases, in contrast to the predominantly sporadic nature of the disease. To date, four distinct subtypes of familial PA exhibiting Mendelian inheritance patterns, transmitted as autosomal dominant traits, have been identified (67, 87–89). **Table 3** summarizes the currently known genetic abnormalities and their corresponding mechanisms that contribute to autonomous aldosterone production. Historically, familial hyperaldosteronism (FH) has been categorized into type I and type II, with FH type II defined as a heritable form of PA in which FH type I has been excluded. However, the identification of additional causative genetic mutations has prompted further subclassification of FH type II into distinct genetic subtypes. These forms of PA have a young onset age and are frequently diagnosed during early childhood. Individuals diagnosed with PA below the age of 20 or who have multiple family members with PA should be evaluated (104).

**Table 3:** Familial forms of PA.

Subtype of PA	Genetic variant and encoded protein	Brief description and clinical features
FH Type I (FH-I) Chimeric gene	*CYP11B1/CYP11B2* hybrid gene *CYP11B2*	Fusion of an unequal crossing over of highly homologous *CYP11B2* and *CYP11B1*, and formed chimeric gene duplications, resulting in ectopic expression of *CYP11B2* in ZF under the regulation of ACTH. Presents with elevated production of hybrid steroids (18-hydroxycortisol and 18-oxocortisol) measured in urine (90). Severe hypertension (>180/120 mmHg) was usually found in subjects with young onset of hypertension (age <15 years old) and was more frequently in male patients (91). A glucocorticoid, which suppresses ACTH and inhibits aldosterone production, is given to remit PA (92).
FH Type II (FH-II) Germline *CLCN2* mutation	*CLCN2* mutations CIC^-2^ (chloride voltage-gated channel 2)	Occur with varying phenotypes. Clinically and biochemically similar to sporadic forms of PA (93, 94). Link to chromosome region 7p22 established in some families (95). A gain-of-function mutation in Cl^−^ channels leads to ZG depolarization and increased cytoplasmic Ca^2+^ concentration and thus induces aldosterone production (96). Early-onset PA, usually before age 20 years. Incomplete penetrance and heterogeneity in phenotype expression. Positive response to spironolactone treatment (96).
FH Type III (FH-III) Germline *KCNJ5* mutation	*KCNJ5* mutations GIRK4 (potassium voltage-gated channel subfamily J member 5)	Mutation in the G-protein-activated inward rectifier potassium channel GIRK4 leads to loss of ZG membrane potential and increase in influx of Na^+^ into the cell, thus triggering overexpression of *CYP11B2* and aldosterone overproduction (97). Presents with elevated production of 18-hydroxycortisol and 18-oxocortisol (98, 99). Early-onset severe hypertension in childhood, before age 10 years old. Presents with ZF hyperplasia and ZG atrophy. Non-suppressive aldosterone with glucocorticoid treatment, achieved complete cure after adrenalectomy (98).
FH Type IV (FH-IV) Germline *CACNA1H* mutation	*CACNA1H* mutations Ca_v_3.2 (calcium voltage-gated channel subunit α1 H)	Mutation in voltage-gated calcium channel (Ca_v_3.2) leads to ZG membrane depolarization, increased intracellular Ca^2+^ influx, and thus increased aldosterone production (100). Early-onset severe hypertension in childhood, before age 10 years old. Incomplete penetrance, heterogeneity in phenotype expression with some asymptomatic patients (100).. Presents with other clinical features: neurologic abnormalities, epilepsy (101), autism (102), and chronic pain (103).
Germline *CACNA1D* mutation	*CACNA1D* mutations Ca_v_1.3 (calcium voltage-gated channel subunit α1 D)	Mutation in the α1 subunit of the L-type voltage-gated calcium channel Ca_v_1.3, activating Ca^2+^ at reduced depolarization potentials, leading to increased Ca^2+^ influx and overexpression of CYP11B2 (67). Early onset, at birth, or usually before age 10 years old. Featuring neuromuscular abnormalities (PASNA—Primary Aldosteronism associated with Seizures and Neurologic Abnormalities) (67).

Last but not least, the exceedingly rare APACCs account for less than 1% of all PA cases. Aldosterone overproduction is often co-secreted with other steroids, such as glucocorticoids, estrogens, or androgens, leading to the co-presentation of Cushing’s syndrome, virilization, or feminization (105–107). Clinicians should maintain a high index of suspicion for adrenocortical carcinoma (ACC) during PA screening, even when an adrenal mass is detected without definitive malignant features on CT imaging (108, 109). ACCs, though rare, are highly malignant and require prompt diagnosis and treatment. The presence of a mass larger than 40 mm, with irregular borders, heterogeneous density, and rapid growth, should raise suspicion for malignancy, even if other features are not definitively diagnostic.

## 7 Genetic mechanisms and clinical characteristics underlying sporadic primary aldosteronism

Choi et al. (2011) were the first to report somatic mutations associated with APAs (66). Two recurrent mutations, *KCNJ5* G151R and L168R, were identified, and both resulted in alterations in ion channel function, especially an abnormal increase in Na^+^ permeability of the cell membrane, leading to an increase in aldosterone production. The study also reported an inherited *KCNJ5* mutation (T158A) that presented with severe aldosteronism and massive bilateral adrenal hyperplasia. These findings highlight the significant role of ion channel mutations in the pathophysiology of PA, providing crucial insights into the molecular mechanisms that drive abnormal secretion of aldosterone. The *KCNJ5* gene is the most frequently implicated in APA, with mutations observed in more than 40% of cases (78). The prevalence of *KCNJ5* mutations is even higher in cohorts from Japan and other East Asian regions, reaching approximately 65%–69% (110–112). These mutations are notably more common in female patients (accounting for over 70%) and younger individuals, often presenting with larger tumor sizes. Furthermore, patients with *KCNJ5* mutations typically exhibit elevated preoperative aldosterone levels and lower serum potassium levels, which may contribute to the early onset of the disease, its increased severity, and earlier diagnosis (78–81).

The *CACNAID* mutations are the second most prevalent somatic mutations reported in APAs, occurring in 9%–27% of cases, with significant male and Black ethnicity dominance (78, 82, 113). It encodes a voltage calcium channel that contains four homologous repeats (I–IV), each with six transmembrane segments (S1–S6). Scholl et al. (2013) found *CACNA1D* G403R and I770M somatic mutations in 5 of 43 APAs without *KCNJ5* or *CTNNB1* mutations (67). These modified residues (*CACNA1D* G403R and *CACNA1D* I770M) are located in the S6 segments that form the channel pores. Changes in both positions led to channel activation at lower depolarized potentials, and modifications at the Gly403 position also disrupted channel inactivation. These mutations caused a shift in voltage-dependent gating towards more negative voltages, reduced inactivation, and enhanced currents (68). These changes lead to an increase in Ca^2+^ influx, which is thought to stimulate aldosterone production and ZG cell proliferation (67). *CACNA1D* mutant APMs tend to be smaller than *KCNJ5* mutant APAs, with many of these being <10 mm, which could have been overlooked with conventional adrenal imaging (68, 78). Scholl et al. (2013) also found these mutations to be *de novo* germline mutations in two children with PA and neuromuscular abnormalities (67).

Following the discovery of *KCNJ5* and *CACNA1D* mutations in APA, two members of the P-type ATPase gene family, *ATP1A1* (encoding Na^+^/K^+^-ATPase I) and *ATP2B3* (encoding Ca^2+^-ATPase 3) mutations were reported in 5.2% (16 subjects) and 1.6% (5 subjects) of cases, respectively, from a total of 308 subjects with APA screened by Beuschlein et al. (2013) (69). These ATPases are expressed in adrenal cells and play a critical role in regulating sodium, potassium, and calcium ion homeostasis. The prevalence of aldosterone-driving ATPases mutations in APA is low compared to that of *KCNJ5* mutations, approximately 1%–6%, with a higher prevalence in Western cohorts and male dominance (83). Subjects harboring ATPase mutations clinically showed increased aldosterone production and severe hypokalemia with smaller tumor sizes (69, 114).

Similar to *KCNJ5* and *CACNA1D* aldosterone-driving mutations, *CLCN2* and *CACNA1H* aldosterone-driving mutations have been previously reported as germline mutations associated with FH. In 2018, exome sequencing of an APA from a female patient with early-onset PA diagnosed at age 9 years identified a *G24D* somatic mutation situated in a well-conserved domain of the *CLCN2* gene encoding the voltage-gated chloride channel (72, 115, 116). The *CLCN2* G24D variant caused inactivation of the channel, increasing the Cl^−^ current by eliminating the voltage of CIC-2 at resting potentials, thus resulting in increased *CYP11B2* expression and aldosterone production. Likewise, exome sequencing of 40 unrelated individuals diagnosed with PA and hypertension by the age of 10 years identified five cases (12.5%) carrying the same *CACNA1H* (M1549V) mutation. Two of the cases arose *de novo*, and all were found to have occurred independently (100). *CACNA1H* encodes the voltage-gated T-type calcium channel alpha subunit Ca_v_3.2. However, the aldosterone-driving mutation *CACNA1H* I1430T was identified in APA using the *CYP11B2*-guided sequencing approach (73). All variants affected intracellular calcium signaling, similar to other somatic mutations involving ion channels or ion transporters reported in PA. The discovery of mutations in early-onset PA and APA indicates that these susceptibility genes may act across diverse phenotypes.

In contrast, the role of *CTNNB1* mutations in APAs is believed to be tumor developer rather than aldosterone stimulator. The gene *CTNNB1*, encoding the protein β-catenin located on human chromosome 3p21~22, with a total length of 23.2 kb and 16 exons, is the core molecule for the Wnt/β-catenin signaling pathway (117, 118). The Wnt/β-catenin signaling pathway is essential for maintaining normal cellular growth of the adrenal cortex, particularly the ZG, regulating cellular proliferation and differentiation (119–121). Simultaneously, this signaling pathway contributes to both the initiation and progression of tumor formation. Aberrant activation of gene transcription by β-catenin is frequently found in human cancers and adrenal adenomas (70, 121, 122). The *CTNNB1* S37C and *CTNNB1* S45F mutations (first reported to be related to APA) inhibit the phosphorylation of β-catenin, resulting in the abnormal activation of the Wnt/β-catenin signaling pathway (70). Interestingly, Teo et al. (2015) found three cases harboring *CTNNB1* mutations that presented with hyperaldosteronism during either pregnancy or menopause, and expressing *LHCGR* and *GNRHR* at levels 100 times higher than those found in other APAs. This phenomenon is thought to result from Wnt/β-catenin pathway activation and the consequent dedifferentiation of adrenal cortical cells, permitting the ectopic expression of gonadal hormone receptors (71). However, *CTNNB1* mutations are not exclusive to female patients, as evidenced by their presence in male patients as well (123, 124). Furthermore, elevated *LHCGR* and *GNRHR* expression has been detected in approximately 45% of APAs, suggesting a more complex underlying pathophysiology than initially proposed (125–127). Further clarifying, the whole-exome sequencing results of 41 unrelated APAs had identified gain-of-function somatic mutation in *CTNNB1* co-existing with *GNA11/GNAQ* mutations at position Q209 in three subjects (74). Subsequent genotyping performed by the group further identified these double mutants in 16 subjects (achieved 100% complete clinical success after adrenalectomy), 15 of whom were women who also presented with an elevation of *LHCGR* expression, most of whom presented with PA during the first trimester of pregnancy (74).

Whole-exome sequencing of APAs also discovered *CADM1* (encoding cell adhesion molecule 1) G379D and V380D somatic mutations in two hypertensive subjects with periodic presentation of PA, who were completely cured post-adrenalectomy (75). The *CADM1* mutations inhibit gap junction intercellular communication between aldosterone-producing cells. Interestingly, transduction of the mutations into human adrenocortical H295R cells increased not only *CYP11B2* expression but also differentially expressed genes associated with biological rhythm processes (75). In the same year (2023), somatic mutations in *SLC30A1* (encoding the zinc efflux transporter zinc transporter 1, ZnT1) L51_A57del and L49_55del variants were reported in three and two subjects with APA, respectively (76). Functional studies of the *SLC30A1* 51_57del variant in the HAC15 human adrenocortical cell line with doxycycline stimulation showed that this variant caused abnormal Na^+^ conductivity, depolarization of the resting membrane potential, and opening of voltage-gated calcium channels. Thus, increasing the cytosolic Ca^2+^ activity led to the elevation of *CYP11B2* mRNA expression and aldosterone production (76).

The most recent recurrent somatic mutation in APA reported is within the *MCOLN3* gene, which encodes the transient receptor potential cation channel mucolipin-3 (TRPML3) (77). Two *MCOLN3* variants, Y391D and N411_V412delinsI, were identified in adrenal tumors resected from three male subjects, with tumor sizes ranging from 12 to 17 mm. The subject harboring the *MCOLN3* N411_V412delinsI mutation presented with more pronounced clinical features, including severe hypertension and hypokalemia, compared to those with the *MCOLN3* Y391D missense mutation. Aberrant *MCOLN3* expression has been shown to disrupt intracellular homeostasis, thereby promoting autonomous aldosterone production. Rooyen et al. proposed two potential mechanisms by which the *MCOLN3* Y391D variant may lead to dysregulated aldosterone production, either through direct enhancement of Ca^2+^ influx or indirectly via altered ion selectivity, causing membrane depolarization and subsequent Na^+^ and then Ca^2+^ influx. Interestingly, although *MCOLN3* is abundantly expressed in the adrenal cortex and APA tumors, its expression does not appear to correlate with *CYP11B2* expression, implying that these genes are subject to independent regulatory pathways (77).

## 8 Effect of aldosterone-driving mutation on adrenal cell fate

The impact of APA mutations on aldosterone overproduction has been well established over the past decade. However, their potential role in dysregulating the mechanisms that control adrenal cell proliferation, thereby promoting adrenal cell mass expansion, tumorigenesis, and APA formation, remains incompletely understood. Current understanding of APA tumorigenesis is based on two models: (i) the two-hit model (128) and (ii) the APM model (129). The two-hit model suggests that during APA formation, the first hit causes increased cell proliferation driven by genetic or environmental factors, followed by the second hit, which is due to the occurrence of APA somatic mutation. A case report by Vouillarmet et al. (2016) of a young bilateral macronodular adrenal hyperplasia patient due to familial adenomatous polyposis supports this two-hit model. Histological examination of the resected adrenal gland revealed three predominantly nonfunctional nodules, all harboring a germline heterozygous mutation in the adenomatous polyposis coli (APC, also known as deleted in polyposis 2.5) encoded by the *APC* gene. Notably, only the nodule with detectable *CYP11B2* expression harbored an additional somatic mutation in *KCNJ5*. These findings suggest that the *APC* mutation may confer a predisposition for adrenal cortical remodeling characterized by elevated nodulation and reduced vascularization, whereas the somatic *KCNJ5* mutation appears to be specifically associated with autonomous aldosterone production (130).

The alternative APM model proposed is that the accumulation of somatic mutation alterations in ZG cells leads to the formation of an APM, which then progresses into a nodule and finally transforms into an APA. Of note, overexpression of *KCNJ5* mutants causes cell death rather than proliferation in HAC15 cells, which is likely due to massive Na^+^ influx (97, 131). This finding seems to be at odds with *in vivo* data, which reports that APAs harboring a *KCNJ5* mutation are generally larger compared to APA *KCNJ5* wild type (132, 133). Moreover, the adrenal cortex hyperplasia phenotype presenting in patients with germline *KCNJ5* mutation indicates that these mutations may contribute to tumorigenesis by enhancing cell proliferation or inhibiting trans-differentiation processes *in vivo* (97, 134, 135). Adrenal hyperplasia in a patient with germline mosaicism localized to areas with *KCNJ5* mutations further supports this hypothesis (134). These conflicting *in vitro* and *in vivo* results, however, could perhaps be in harmony with each other when taking into account Yang et al.’s finding that the effect of *KCNJ5* mutation on cell proliferation is impacted by the expression levels of the mutated channel (i.e., at low levels of expression, no cell deaths occur) (131).

## 9 Conclusion

The adrenal gland plays a central role in endocrine regulation, with aldosterone synthesis being essential for maintaining cardiovascular stability and electrolyte balance. Advances in the molecular characterization of PA have uncovered an expanding spectrum of somatic and germline mutations that disrupt ion transport, calcium signaling, and adrenal cortical differentiation. These findings not only elucidate the mechanisms driving aldosterone hypersecretion but also reveal diverse pathways involved in adrenal tumorigenesis and altered cell fate. Understanding these molecular determinants could pave the way for more precise diagnostic frameworks and mutation-informed therapeutic strategies in PA. Pharmacogenomic approaches that incorporate individual genomic profiles have the potential to refine diagnosis, predict treatment response, and guide personalized management. Moreover, mutation-specific therapeutic targets, beyond the current focus on CYP11B2 inhibition, also present promising avenues for tailored interventions. Continued integration of genetic, cellular, and clinical insights will be essential to advance precision medicine in PA, ultimately enabling earlier detection, individualized therapy, and improved long-term cardiovascular outcomes.
